# Occupational distribution of metabolic syndrome prevalence and incidence differs by sex and is not explained by age and health behavior: results from 75 000 Dutch workers from 40 occupational groups

**DOI:** 10.1136/bmjdrc-2020-001436

**Published:** 2020-07-06

**Authors:** Sander K R van Zon, Benjamin C Amick III, Trynke de Jong, Sandra Brouwer, Ute Bültmann

**Affiliations:** 1 Department of Health Sciences, Community and Occupational Medicine, University of Groningen, University Medical Center Groningen, Groningen, The Netherlands; 2 Department of Epidemiology, University of Arkansas for Medical Sciences Fay W Boozman College of Public Health, Little Rock, Arkansas, USA; 3 Lifelines Cohort and Biobank Study, Roden, The Netherlands

**Keywords:** epidemiology, public health, metabolic syndrome, occupational health

## Abstract

**Introduction:**

This study examines the association between 40 occupational groups and prevalence and incidence of metabolic syndrome (MetS), separately for male and female workers, and whether age and health behaviors can explain the association.

**Research design and methods:**

Data from 74 857 Lifelines Cohort and Biobank Study participants were used to regress occupational group membership, coded by Statistics Netherlands, on the prevalence and incidence of MetS using logistic and Cox regression analyses. MetS diagnosis was based on physical examinations, blood analysis, and recorded medication use. Information on age, smoking status, physical activity, diet and alcohol consumption was acquired using questionnaires.

**Results:**

Baseline MetS prevalence was 17.5% for males and 10.6% for females. During a median 3.8 years of follow-up, MetS incidence was 7.8% for males and 13.2% for females. One occupational group was associated with an increased MetS risk in both sexes. Six additional occupational groups had an increased risk for MetS among men, four among women. Highest risks were found for male ‘stationary plant and machine operators’ (HR: 1.94; 95% CI 1.26 to 3.00) and female ‘food preparation assistants’ (HR: 1.80; 95% CI 1.01 to 3.22).

**Conclusions:**

Findings suggest that occupational group matters for men and women in MetS development, and that differences in MetS prevalence across occupations are not merely a reflection of selection of metabolically unhealthy workers into specific occupations. The striking sex differences in the occupational distribution of MetS indicate that preventive measures should, with some exceptions, target men and women separately.

**Significance of this study**

**What is already known about this subject?**

- Occupational group membership is associated with metabolic syndrome prevalence.
- Age and health behaviors are associated with metabolic syndrome.

**What are the new findings?**

- Occupational group membership is associated with metabolic syndrome incidence.
- Age and health behaviors do not account for the increased incidence in all occupational groups.
- There are striking sex differences in the occupational distribution of metabolic syndrome incidence.

**How might these results change the focus of research or clinical practice?**

- The striking sex differences in the occupational distribution of metabolic syndrome indicate that preventive measures should, with some exceptions, target male and female workers separately.
- Researcher should further investigate how the workplace can be optimized as a setting for health promotion and prevention of cardiometabolic conditions like metabolic syndrome.

## Introduction

Metabolic syndrome (MetS), a cluster of preclinical conditions (ie, central obesity, insulin resistance, hypertension, raised triglycerides and lowered high-density lipoprotein (HDL) cholesterol),^1^ doubles the risk of cardiovascular outcomes and more than triples the risk for type 2 diabetes mellitus (T2DM).^2 3^ In addition, the average annual healthcare costs are 1.6 times higher for people with MetS than for people without MetS.^4^ The global prevalence of MetS is currently about 25%, which means that over a billion people are affected.^5^ The prevalence of MetS increases with age and prevalence estimates in Europe range from 4% among 20–29 year-olds to almost 30% among 60–69 year-olds.^6^

The proportion of working-age individuals with MetS underscores the need to understand the role of work in contributing to the population distribution of MetS.^6^ An important step in examining the role of work in MetS is to surveil the occupational distribution of MetS. To date, studies have shown that occupational groups that require lower skill levels, like ‘machine installers, operators, and assemblers’,^7 8^ ‘construction workers’,^9^ and ‘food preparation occupations’,^10^ have the highest prevalence of MetS. Studies on components of MetS found similar occupations like ‘motor vehicle operators’, ‘transportation workers’ but also ‘protective services’ to be associated with high rates of obesity^11–14^ and hypertension.^15 16^

Yet, current evidence is limited in at least two ways. First, studies on MetS are cross-sectional and therefore do not clarify whether occupational group membership contributes to the development of MetS or differences are merely a reflection of selection of metabolically unhealthy workers into specific occupational groups.^7–10^ Second, studies did not adjust the relationship between occupational groups and MetS for smoking, physical activity, diet and alcohol consumption^7 9^—health behaviors known to predict MetS,^17^ or did so with limited information on occupational group membership (ie, adjusted analyses with only five or 13 occupational groups).^8 10^

Furthermore, the occupational distribution of MetS is likely to differ between men and women for several reasons. Certainly, men and women have different work experiences and therefore any assessment of the role of work must examine population-based sex differences by occupation. In addition, the etiology of MetS and its components, and the prevalence of MetS, differ between men and women.^18–21^ Most noticeably, sex hormonal influences differ in men and women and could explain sex differences in the etiology and prevalence of MetS.^18 20^ Sex-stratified analyses are therefore needed when investigating MetS, especially in the context of work.^21^ Studies assessing the sex-specific occupational distribution of MetS find remarkable differences between men and women.^7 9^ Most notably, MetS prevalence is thrice as high among males in high-end jobs like ‘general managers and government administrators’ than among their female counterparts.^7^

Against this background, three research questions about MetS prevalence and incidence rates using a sex-specific approach are addressed. First, are occupational groups associated with MetS? Second, are associations of occupational group and MetS due to the age distributions across occupational groups? Third, are the associations of occupational group and MetS influenced by smoking, physical activity, diet and alcohol consumption—health behaviors known to predict MetS?

## Research design and methods

### Study design and sample

The study was conducted using data from the longitudinal Lifelines Cohort Study.^22^ Between 2007 and 2013, a total of 167 729 persons living in the northern part of the Netherlands were recruited through general practitioners, family members and self-registry. At baseline, participants visited one of the Lifelines research centers for a physical examination and collection of biological samples. Participants further completed extensive questionnaires. In total, 152 728 adult participants were included at baseline. Participants received follow-up questionnaires after approximately 1.5, 3 and 5 years. After 5 years, participants also revisited one of the Lifelines research centers for a physical examination and collection of biological samples. For the current study, we selected participants of working age (ie, 18–64 years old), who were part of the working population according to the Dutch and international definition (ie, working at least 1 hour per week),^23^ and who completed the first and second physical examinations. With these restrictions, 12 689 of 152 728 participants were excluded due to age ineligibility, 22 742 participants were excluded because they were not working, and 40 563 participants were excluded because they had no follow-up measurement (ie, did not yet complete the measurement or lost to follow-up).

### Measures and procedures

#### Occupational group membership

Occupational group membership was assessed at baseline by asking participants about their occupation and the main tasks related to their occupation. Statistics Netherlands coded all occupations automatically according to the International Standard Classification of Occupations (ISCO) 08.^24^ We performed a quality control by selecting a 1% subsample of the complete baseline data set (n=1432). Statistics Netherlands provided a certainty score (range 0–100), indicating how well occupations could be coded based on the information participants provided. We examined possible misclassification regardless of the certainty score and for different cut-off values (ie, certainty scores ≥50, ≥60, ≥70, and ≥80).

Forty of the 43 submajor occupational groups (ie, two-digit level) were included in our analyses.^25^ Three submajor occupational groups belonging to the major occupational group ‘armed forces’ were excluded because the number of participants in this major occupational group was very small (n=129). We also explored a more refined grouping of occupational groups (three-digit codes) but the sample size was too small in many occupational groups. We had no ISCO classification for 1748 participants (ie, missing data) so they were excluded from the analytical study sample. The selection of the analytical study sample is shown in online supplementary figure 1.

10.1136/bmjdrc-2020-001436.supp1 Supplementary data

The quality control showed that at least 81.2% of the occupational classifications were correct at the submajor group level when the certainty level was not taken into account (online supplementary table 1). Misclassification decreased with an increasing certainty score, with 92.7% agreement on the submajor group level when the certainty score was ≥60. However, 44.7% of participants in the total sample had a score <60. The proportion of participants with a certainty score <60 was largest among managers (68.6%) (online supplementary table 2).

10.1136/bmjdrc-2020-001436.supp2 Supplementary data

#### Metabolic syndrome

MetS was defined according to the joint interim criteria.^1^ The diagnosis MetS was established if at least three of the following five components were present: (1) central obesity (waist circumference (WC) ≥102 cm in men, WC ≥88 in women), (2) raised triglycerides (≥1.7 mmol/L) or treatment for this lipid abnormality, (3) reduced HDL cholesterol (<1.0 mmol/L in men, <1.3 mmol/L in women) or treatment for this lipid abnormality, (4) elevated blood pressure (systolic blood pressure (SBP) ≥130 mm Hg and/or diastolic blood pressure (DBP) ≥85 mm Hg) or treatment of previously diagnosed hypertension, (5) raised fasting plasma glucose (FPG) (≥5.6 mmol/L) or previously diagnosed T2DM. *WC* was measured in an upright position and in the middle between the front end of the lower ribs and the iliac crest. *Triglycerides* and *HDL cholesterol* were determined based on fasting blood samples. Treatment for lipid abnormality, that is, cholesterol-lowering medication (ie, Anatomical Therapeutic Chemical (ATC) codes C10A, C10B), was recorded at baseline only.^26^ Mean SBP and DBP were measured using an automatic blood pressure monitor.^22^ Antihypertensive medication (ie, ATC codes C02, C03, C07, C08, C09) was recorded only at baseline.^26^ *FPG* was determined based on fasting blood samples. Previously diagnosed T2DM was based on medication use (ie, ATC codes A10A, A10B) only at baseline.^26^ Physical measurements and blood samples were taken by trained research staff using standardized protocols and calibrated measuring equipment.^22^

#### Sociodemographic factors and health behavior

Baseline sociodemographic factors and health behaviors included age, gender, educational level, smoking status, alcohol consumption, physical activity, and dietary habits and were, except for dietary habits, coded in accordance with previous studies using Lifelines data.^27^ Dietary habits were coded based on the Dutch guideline for healthy nutrition.^28^ *Educational level* was categorized into low, medium and high. *Smoking status* was categorized as being a current smoker, ex-smoker, or never smoker. *Alcohol consumption* was categorized into drinking 0 days/week, drinking 0–1 days/week, drinking >1 to 3 days/week and drinking >3 days/week. *Physical activity* was based on the number of days per week participants were active for at least half an hour (eg, bicycle, exercise) and was categorized into being inactive (0–2 days per week), moderately active (3–4 days per week), or active (≥5 day per week). *Diet* was based on fruit and vegetable consumption. Participants eating both fruits and vegetables ≥4 days per week were categorized as having a healthy diet, participants eating fruits ≥4 days per week but vegetables <4 days per week, or vice versa, were categorized as having a moderately healthy diet, and participants eating both fruits and vegetables <4 days per week were categorized as having an unhealthy diet.

#### Statistical analyses

First, the distribution of baseline sociodemographic and health behavior factors was examined for the total population and separately for men and women. Differences between participants excluded for missing follow-up data (n=40 563) and those included were examined to assess possible bias. Second, the association between baseline MetS prevalence and submajor occupational groups was estimated using sex-specific logistic regression analyses. Third, the risk of 5-year incidence of MetS by submajor occupational group was estimated using sex-specific Cox regression analyses. Both cross-sectional and longitudinal analyses were, after crude analyses (model 1), adjusted for age (model 2), and smoking, physical activity, diet, and alcohol consumption (model 3) to understand the unique contribution of occupational group in determining the MetS population distribution. Educational level is not adjusted for because it conceptually overlaps with occupational group membership. These two factors were moderately correlated in our data set (Spearman’s correlation: 0.56). ‘Science and engineering professionals’ were the reference category because they have a high occupational skill level and the certainty score for the occupational coding of ‘professionals’ was considerably higher than for ‘managers’, who also have a high occupational skill level (online supplementary table 2).

## Results

### Baseline characteristics

In total, 31 969 men and 42 888 women were included in the study (table 1). The mean age was 43.0 years (SD: 10.0) for men and 42.1 years (10.2) for women. Baseline MetS prevalence was 17.5% (5590/31 969) for men and 10.6% (4540/42 888) for women. The baseline prevalence of central obesity and reduced HDL cholesterol were higher among women while raised triglycerides, elevated blood pressure and raised FPG were more common among men. Baseline characteristics of participants with and without follow-up data differed for some variables, but differences were generally small (online supplementary table 3). Baseline study participants without follow-up data were younger, had poorer health behavior, and had higher rates of reduced HDL and increased glucose levels, but lower rates of raised blood pressure and MetS. The median follow time was 3.8 years (IQR: 3.1–4.7 for men; 3.1–4.6 for women) and 7.8% (2068/26 379) of men and 13.2% (5044/38 348) of women developed MetS, respectively.

**Table 1:** Baseline characteristics for the total study sample and for men and women

	Total		Men		Women	
	n	% or mean (SD)	n	% or mean (SD)	n	% or mean (SD)
**Sociodemographic factors**						
Age (years)	74 857	42.5 (10.1)	31 969	43.0 (10.0)	42 888	42.1 (10.2)
Educational level	73 713		31 598		42 115	
High		34.0		34.8		33.4
Medium		42.4		40.0		44.2
Low		23.6		25.2		22.4
**Health behavior**						
Smoking status	70 807		30 240		40 567	
Non-smoker		47.9		46.7		48.9
Former smoker		31.0		30.2		31.5
Current smoker		21.1		23.1		19.6
Alcohol consumption	74 269		31 723		42 546	
0 days/week		19.0		9.2		26.3
0–1 days/week		19.8		15.2		23.2
1–3 days/week		40.6		49.1		34.4
>3 days/week		20.6		26.6		16.1
Physical activity	71 939		30 828		41 111	
High		46.3		44.7		47.4
Moderate		27.0		27.6		26.4
Low		26.8		27.6		26.2
Diet	74 258		31 713		42 545	
Healthy		21.2		14.7		26.0
Moderate		66.7		69.1		64.8
Unhealthy		12.2		16.2		9.1
**Health**						
Central obesity	74 846	31.9	31 965	22.6	42 881	38.9
Raised triglycerides	74 329	17.1	31 802	27.3	42 527	9.5
Reduced HDL cholesterol	74 329	16.8	31 802	14.6	42 527	18.4
Raised blood pressure	74 832	38.4	31 961	52.3	42 871	28.0
Raised fasting plasma glucose	74 022	10.5	31 661	15.2	42 361	6.9
Metabolic syndrome	74 857	13.5	31 969	17.5	42 888	10.6

HDL, high-density lipoprotein.

### MetS prevalence by occupational group

MetS prevalence across occupational groups was generally higher for men than women (online supplementary figure 2). Among men, ‘drivers and mobile plant operators’ (25.7%) and ‘hospitality, retail and other service managers’ (25.2%) had the highest rates of MetS while the prevalence among women was highest for ‘assemblers’ (21.7%) and ‘drivers and mobile plant operators’ (20.0%). Online supplementary table 4 show the stepwise-adjusted associations between the occupational groups and MetS for men. In the unadjusted model (model 1), 14 occupational groups were associated with an increased odds for MetS, which was reduced to 11 after adjustment for age (model 2), and 6 after adjustment for health behavior (model 3). For men, ‘hospitality, retail and other service managers’ (OR: 1.65; 95% CI 1.03 to 2.65) and ‘drivers and mobile plant operators’ (OR: 1.57; 95% CI 1.28 to 1.93) had the highest ORs for MetS. Online supplementary table 5 shows the stepwise-adjusted associations for women. In the unadjusted model, 23 occupational groups were associated with an increased odds for MetS. The number with increased odds decreased to 18 after adjustment for age and 12 after adjustment for health behavior. ‘Stationary plant and machine operators’ (OR: 3.44; 95% CI 1.57 to 4.54) and ‘assemblers’ (OR: 3.43; 95% CI 1.26 to 9.33) had the highest odds for MetS. Only three occupational groups were associated with an increased odds for MetS in both men and women (ie, ‘customer services clerks’, ‘stationary plant and machine operators’, and ‘drivers and mobile plant operators’). All other associations were sex specific.

### MetS incidence by occupational group

**Figure 1:**
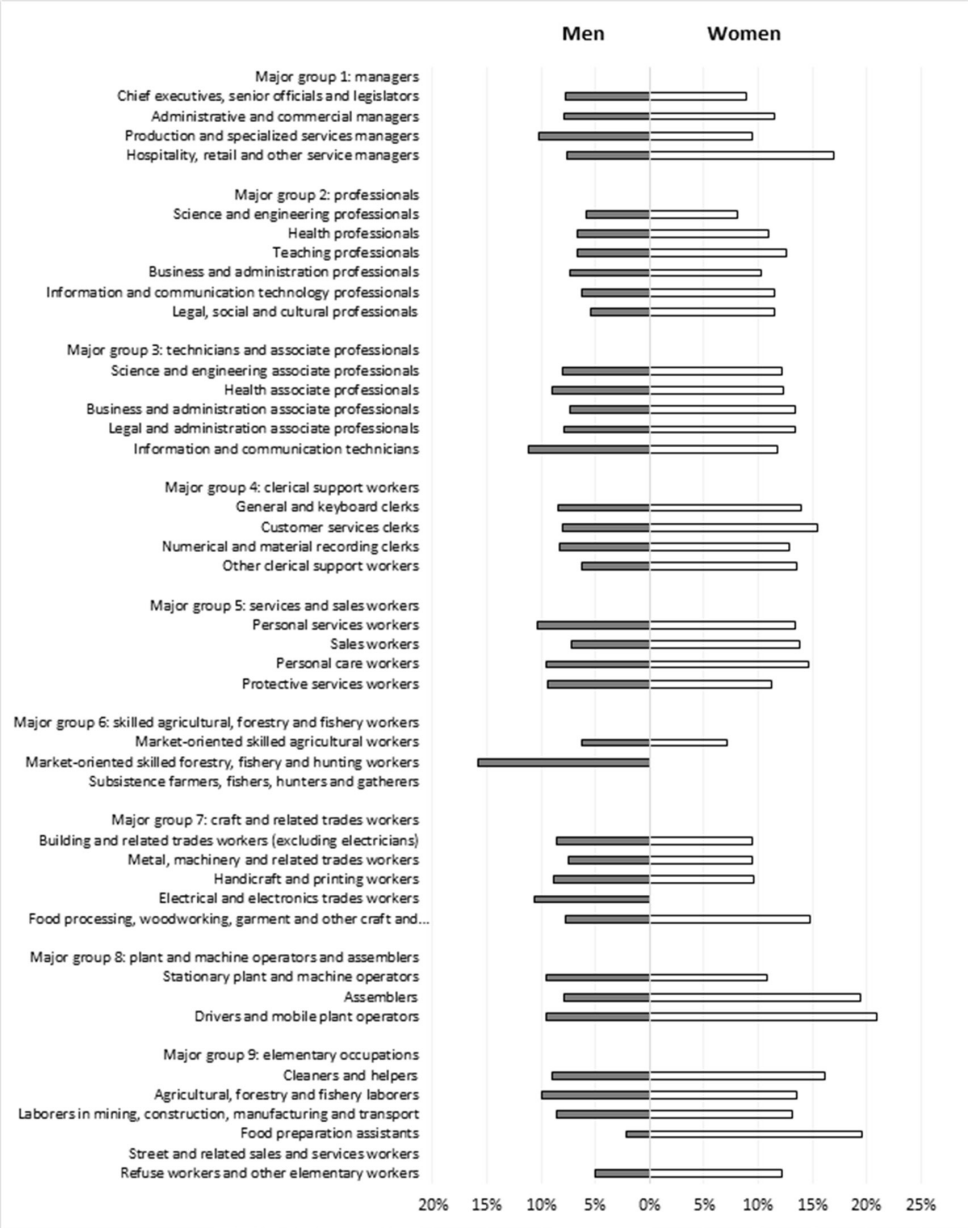
Incidence of metabolic syndrome stratified for men and women.

In contrast to baseline prevalence, MetS incidence was generally higher for women than men across the occupational groups (figure 1). Among men, ‘market-oriented skilled forestry, fishery and hunting workers’ (15.8%) and ‘information and communication technicians’ (11.2%) had the highest MetS rates. Among women, MetS incidence was highest for ‘drivers and mobile plant operators’ (20.9%) and ‘food preparation assistants’ (19.6%). Table 2 shows the stepwise-adjusted risks for MetS by occupational group for men. In the unadjusted model (model 1), 14 occupational groups had an increased risk for MetS, which remained unchanged after adjustment for age (model 2) and was reduced to 7 after adjustment for health behavior (model 3). ‘Stationary plant and machine operators’ (HR: 1.94; 95% CI 1.26 to 3.00) and ‘electrical and electronics trades workers’ (HR: 1.83; 95% CI 1.25 to 2.68) had the highest HRs.

**Table 2:** The incidence of metabolic syndrome among men, and its association with submajor occupational groups

		N MetS/n total	%	Model 1	Model 2	Model 3
				HR (95% CI)	HR (95% CI)	HR (95% CI)
	**Major group 2: professionals**					
21	Science and engineering professionals	70/1204	5.8	Ref	Ref	Ref
22	Health professionals	41/610	6.7	1.12 (0.76 to 1.64)	1.04 (0.71 to 1.53)	1.08 (0.72 to 1.63)
23	Teaching professionals	72/1098	6.6	1.04 (0.75 to 1.45)	0.91 (0.65 to 1.26)	1.02 (0.72 to 1.43)
24	Business and administration professionals	158/2121	7.4	1.22 (0.92 to 1.62)	1.19 (0.89 to 1.57)	1.21 (0.90 to 1.62)
25	Information and communication technology professionals	84/1350	6.2	1.05 (0.77 to 1.45)	1.10 (0.80 to 1.51)	1.12 (0.80 to 1.56)
26	Legal, social and cultural professionals	35/648	5.4	0.91 (0.60 to 1.36)	0.83 (0.55 to 1.24)	0.81 (0.53 to 1.25)
	**Major group 1: managers**					
11	Chief executives, senior officials and legislators	21/274	7.7	1.16 (0.71 to 1.88)	1.00 (0.62 to 1.63)	0.96 (0.58 to 1.61)
12	Administrative and commercial managers	60/764	7.9	1.21 (0.86 to 1.71)	1.16 (0.82 to 1.64)	1.14 (0.79 to 1.63)
13	Production and specialized service managers	69/678	10.2	**1.72 (1.24 to 2.41)**	**1.55 (1.11 to 2.16)**	**1.44 (1.01 to 2.05)**
14	Hospitality, retail and other service managers	7/92	7.6	1.27 (0.59 to 2.77)	1.30 (0.60 to 2.83)	1.10 (0.48 to 2.54)
	**Major group 3: technicians and associate professionals**					
31	Science and engineering associate professionals	122/1511	8.1	**1.38 (1.03 to 1.85)**	**1.35 (1.00 to 1.81)**	1.32 (0.97 to 1.80)
32	Health associate professionals	36/400	9.0	1.44 (0.97 to 2.16)	1.43 (0.95 to 2.13)	1.31 (0.84 to 2.02)
33	Business and administration associate professionals	134/1815	7.4	1.13 (0.85 to 1.51)	1.12 (0.84 to 1.49)	1.13 (0.83 to 1.53)
34	Legal and administration associate professionals	55/700	7.9	1.36 (0.95 to 1.94)	1.33 (0.93 to 1.89)	1.25 (0.85 to 1.82)
35	Information and communication technicians	27/242	11.2	**2.05 (1.32 to 3.20)**	**2.04 (1.31 to 3.18)**	**1.81 (1.11 to 2.94)**
	**Major group 4: clerical support workers**					
41	General and keyboard clerks	15/179	8.4	1.56 (0.89 to 2.72)	1.57 (0.90 to 2.74)	1.55 (0.87 to 2.76)
42	Customer services clerks	30/369	8.1	1.38 (0.90 to 2.12)	1.49 (0.97 to 2.29)	1.54 (0.99 to 2.41)
43	Numerical and material recording clerks	93/1118	8.3	**1.38 (1.01 to 1.88)**	**1.38 (1.01 to 1.89)**	1.35 (0.98 to 1.87)
44	Other clerical support workers	25/397	6.3	1.18 (0.75 to 1.86)	1.12 (0.71 to 1.77)	1.00 (0.62 to 1.63)
	**Major group 5: services and sales workers**					
51	Personal services workers	64/613	10.4	**1.67 (1.19 to 2.34)**	**1.63 (1.16 to 2.29)**	1.37 (0.95 to 1.98)
52	Sales workers	84/1168	7.2	1.19 (0.87 to 1.64)	1.29 (0.94 to 1.77)	1.20 (0.86 to 1.68)
53	Personal care workers	22/228	9.6	**1.74 (1.08 to 2.81)**	**1.72 (1.07 to 2.78)**	1.42 (0.85 to 2.37)
54	Protective services workers	58/615	9.4	**1.69 (1.19 to 2.40)**	**1.61 (1.14 to 2.28)**	**1.59 (1.10 to 2.30)**
	**Major group 6: skilled agricultural, forestry and fishery workers**					
61	Market-oriented skilled agricultural workers	81/1277	6.3	1.11 (0.81 to 1.53)	1.06 (0.77 to 1.46)	1.02 (0.73 to 1.44)
62	Market-oriented skilled forestry, fishery and hunting workers	3/19	15.8	2.06 (0.65 to 6.56)	1.89 (0.60 to 6.01)	1.79 (0.56 to 5.71)
63	Subsistence farmers, fishers, hunters and gatherers	–	–	–	–	–
	**Major group 7: craft and related trade workers**					
71	Building and related trade workers (excluding electricians)	141/1642	8.6	**1.37 (1.03 to 1.83)**	**1.37 (1.03 to 1.83)**	1.27 (0.93 to 1.72)
72	Metal, machinery and related trade workers	94/1255	7.5	1.15 (0.84 to 1.57)	1.17 (0.86 to 1.60)	1.08 (0.77 to 1.49)
73	Handicraft and printing workers	14/160	8.8	1.25 (0.70 to 2.22)	1.24 (0.70 to 2.19)	1.15 (0.63 to 2.09)
74	Electrical and electronics trade workers	49/464	10.6	**1.72 (1.19 to 2.48)**	**1.73 (1.20 to 2.50)**	**1.83 (1.25 to 2.68)**
75	Food processing, woodworking, garment and other craft and related trade workers	37/478	7.7	1.32 (0.89 to 1.97)	1.31 (0.88 to 1.95)	1.09 (0.72 to 1.67)
	**Major group 8: plant and machine operators and assemblers**					
81	Stationary plant and machine operators	31/326	9.5	**1.88 (1.23 to 2.86)**	**1.83 (1.20 to 2.80)**	**1.94 (1.26 to 3.00)**
82	Assemblers	6/76	7.9	1.25 (0.54 to 2.89)	1.38 (0.60 to 3.18)	1.32 (0.57 to 3.06)
83	Drivers and mobile plant operators	102/1065	9.6	**1.69 (1.24 to 2.29)**	**1.63 (1.20 to 2.21)**	**1.44 (1.04 to 1.98)**
	**Major group 9: elementary occupations**					
91	Cleaners and helpers	19/211	9.0	**1.74 (1.05 to 2.88)**	**1.79 (1.08 to 2.97)**	1.47 (0.86 to 2.52)
92	Agricultural, forestry and fishery laborers	5/50	10.0	**2.81 (1.13 to 6.97)**	**2.80 (1.13 to 6.93)**	2.18 (0.79 to 6.01)
93	Laborers in mining, construction, manufacturing and transport	50/582	8.6	**1.45 (1.01 to 2.08)**	**1.63 (1.13 to 2.34)**	**1.63 (1.12 to 2.38)**
94	Food preparation assistants	1/47	2.1	0.39 (0.05 to 2.83)	0.49 (0.07 to 3.50)	0.48 (0.07 to 3.50)
95	Street and related sales and services workers	–	–	–	–	–
96	Refuse workers and other elementary workers	5/101	5.0	0.82 (0.33 to 2.04)	0.84 (0.34 to 2.08)	0.92 (0.37 to 2.28)

Model 1 is unadjusted; model 2 is adjusted for age; model 3 is adjusted for age, smoking, physical activity, diet, and alcohol consumption.

Statistical significant associations are shown in bold.

MetS, metabolic syndrome.

Table 3 shows the stepwise-adjusted risks for MetS by occupational group for women. In the unadjusted model (model 1), 15 occupational groups were associated with an increased risk for MetS, which remained unchanged after adjustment for age and decreased to 5 after adjustment for health behaviors. ‘Food preparation assistants’ (HR: 1.80; 95% CI 1.01 to 3.22) and ‘drivers and mobile plant operators’ (HR: 1.77; 95% CI 1.01 to 3.08) had the highest HRs for MetS. Only one occupational group (ie, ‘drivers and mobile plant operators’) was associated with an increased risk for MetS in both men and women; all others were sex specific.

**Table 3:** The incidence of metabolic syndrome among women, and its association with submajor occupational groups

		N MetS/n total	%	Model 1	Model 2	Model 3
				HR (95% CI)	HR (95% CI)	HR (95% CI)
	**Major group 2: professionals**					
21	Science and engineering professionals	22/273	8.1	Ref	Ref	Ref
22	Health professionals	320/2904	11.0	1.35 (0.87 to 2.07)	1.39 (0.90 to 2.14)	1.29 (0.83 to 2.00)
23	Teaching professionals	384/3050	12.6	**1.54 (1.00 to 2.36)**	**1.61 (1.05 to 2.48)**	1.45 (0.93 to 2.25)
24	Business and administration professionals	187/1808	10.3	1.27 (0.81 to 1.97)	1.30 (0.84 to 2.03)	1.19 (0.46 to 1.88)
25	Information and communication technology professionals	27/235	11.5	1.38 (0.79 to 2.43)	1.42 (0.81 to 2.50)	1.30 (0.73 to 2.32)
26	Legal, social and cultural professionals	148/1282	11.5	1.48 (0.95 to 2.32)	1.52 (0.97 to 2.37)	1.40 (0.88 to 2.21)
	**Major group 1: managers**					
11	Chief executives, senior officials and legislators	11/124	8.9	0.98 (0.48 to 2.03)	1.07 (0.52 to 2.20)	1.02 (0.49 to 2.12)
12	Administrative and commercial managers	44/384	11.5	1.36 (0.81 to 2.27)	1.45 (0.87 to 2.41)	1.20 (0.71 to 2.04)
13	Production and specialized service managers	29/308	9.4	1.02 (0.58 to 1.77)	1.10 (0.63 to 1.92)	1.04 (0.59 to 1.86)
14	Hospitality, retail and other service managers	15/88	17.0	1.79 (0.93 to 3.45)	1.91 (0.99 to 3.68)	1.35 (0.68 to 2.70)
	**Major group 3: technicians and associate professionals**					
31	Science and engineering associate professionals	30/245	12.2	1.45 (0.84 to 2.51)	1.50 (0.86 to 2.60)	1.23 (0.70 to 2.15)
32	Health associate professionals	317/2569	12.3	1.49 (0.97 to 2.30)	1.53 (0.99 to 2.36)	1.32 (0.85 to 2.05)
33	Business and administration associate professionals	360/2682	13.4	**1.65 (1.08 to 2.55)**	**1.71 (1.11 to 2.63)**	1.41 (0.91 to 2.19)
34	Legal and administration associate professionals	343/2561	13.4	**1.67 (1.08 to 2.57)**	**1.71 (1.11 to 2.64)**	1.43 (0.92 to 2.22)
35	Information and communication technicians	11/93	11.8	1.76 (0.85 to 3.62)	1.85 (0.90 to 3.82)	1.36 (0.64 to 2.89)
	**Major group 4: clerical support workers**					
41	General and keyboard clerks	255/1817	14.0	**1.74 (1.13 to 2.69)**	**1.85 (1.19 to 2.85)**	1.50 (0.96 to 2.35)
42	Customer services clerks	206/1330	15.5	**1.92 (1.24 to 2.99)**	**2.02 (1.30 to 3.13)**	**1.66 (1.06 to 2.61)**
43	Numerical and material recording clerks	186/1447	12.9	**1.59 (1.02 to 2.48)**	**1.69 (1.09 to 2.63)**	1.41 (0.89 to 2.22)
44	Other clerical support workers	124/910	13.6	**1.80 (1.14 to 2.83)**	**1.90 (1.21 to 2.99)**	1.53 (0.96 to 2.45)
	**Major group 5: services and sales workers**					
51	Personal services workers	276/2067	13.4	**1.69 (1.09 to 2.61)**	**1.70 (1.10 to 2.63)**	1.38 (0.89 to 2.16)
52	Sales workers	450/3269	13.8	**1.72 (1.12 to 2.64)**	**1.71 (1.11 to 2.62)**	1.35 (0.87 to 2.10)
53	Personal care workers	727/4970	14.6	**1.82 (1.19 to 2.78)**	**1.95 (1.28 to 2.99)**	**1.63 (1.05 to 2.52)**
54	Protective services workers	32/286	11.2	1.41 (0.82 to 2.43)	1.44 (0.84 to 2.48)	1.25 (0.71 to 2.19)
	**Major group 6: skilled agricultural, forestry and fishery workers**					
61	Market-oriented skilled agricultural workers	24/337	7.1	1.06 (0.59 to 1.89)	1.16 (0.65 to 2.07)	1.06 (0.58 to 1.91)
62	Market-oriented skilled forestry, fishery and hunting workers	–	–	–	–	–
63	Subsistence farmers, fishers, hunters and gatherers	–	–	–	–	–
	**Major group 7: craft and related trade workers**					
71	Building and related trade workers (excluding electricians)	6/64	9.4	1.11 (0.45 to 2.74)	1.20 (0.49 to 2.96)	1.12 (0.42 to 2.98)
72	Metal, machinery and related trade workers	4/42	9.5	1.08 (0.37 to 3.12)	1.14 (0.39 to 3.31)	0.93 (0.28 to 3.11)
73	Handicraft and printing workers	7/73	9.6	1.07 (0.46 to 2.52)	1.11 (0.47 to 2.60)	1.23 (0.52 to 2.90)
74	Electrical and electronics trade workers	–	–	–	–	–
75	Food processing, woodworking, garment and other craft and related trade workers	44/297	14.8	**1.97 (1.18 to 3.29)**	**2.04 (1.22 to 3.41)**	1.69 (1.00 to 2.86)
	**Major group 8: plant and machine operators and assemblers**					
81	Stationary plant and machine operators	10/93	10.8	1.23 (0.58 to 2.60)	1.33 (0.63 to 2.80)	1.05 (0.49 to 2.23)
82	Assemblers	7/36	19.4	**3.18 (1.36 to 7.44)**	**3.26 (1.39 to 7.64)**	1.95 (0.79 to 4.83)
83	Drivers and mobile plant operators	36/172	20.9	**2.36 (1.39 to 4.01)**	**2.57 (1.51 to 4.37)**	**1.77 (1.01 to 3.08)**
	**Major group 9: elementary occupations**					
91	Cleaners and helpers	300/1859	16.1	**2.07 (1.34 to 3.19)**	**2.27 (1.47 to 3.51)**	**1.73 (1.11 to 2.70)**
92	Agricultural, forestry and fishery laborers	8/59	13.6	2.00 (0.89 to 4.50)	1.99 (0.89 to 4.48)	1.98 (0.84 to 4.66)
93	Laborers in mining, construction, manufacturing and transport	35/265	13.2	1.65 (0.97 to 2.81)	1.62 (0.95 to 2.77)	1.18 (0.68 to 2.05)
94	Food preparation assistants	28/143	19.6	**2.45 (1.40 to 4.29)**	**2.39 (1.37 to 4.18)**	**1.80 (1.01 to 3.22)**
95	Street and related sales and services workers	–	–	–	–	–
96	Refuse workers and other elementary workers	10/82	12.2	1.64 (0.77 to 3.45)	1.65 (0.78 to 3.49)	1.29 (0.61 to 2.75)

Model 1 is unadjusted; model 2 is adjusted for age; model 3 is adjusted for age, smoking, physical activity, diet, and alcohol consumption.

Statistical significant associations are shown in bold.

MetS, metabolic syndrome.

## Conclusions

In a longitudinal study among 75 000 Dutch workers from 40 submajor occupational groups, MetS prevalence and incidence were associated with occupational group membership. Age and health behaviors do not account for the increased risk in all occupational groups. The occupational groups that matter differ for men and women except the occupational group ‘drivers and mobile plant operators’. In addition, occupational groups associated with MetS vary between prevalence and incidence.

Findings from the current cross-sectional and longitudinal analyses are in line with findings from previous cross-sectional studies on the association between occupational group membership and MetS^7–10^ or components of MetS.^11–16^ Previous studies found that MetS prevalence rates were high for male ‘machine installers, operators, and assemblers’ (15.1%) and for female ‘skilled workers in agricultural and fishing industries’ (8.9%),^7^ and for non-sex specified, ‘food preparation and service occupations’ (31.1%).^10^ Prevalence rates in the current study confirm these findings as male ‘drivers and mobile plant operators’ (25.7%) and female ‘assemblers’ (21.7%) had the highest baseline rates of MetS. Although there are differences in the magnitude of the prevalence between these studies, similar occupational groups showed the highest MetS rates. The incidence rates in the current study showed similar results. Male ‘market-oriented skilled forestry, fishery and hunting workers’ (15.8%) and female ‘drivers and mobile plant operators’ had the highest incidence rates of MetS. Female ‘food preparation assistants’ (19.6%) also had high incident rates of MetS, confirming findings from the USA.^10^

This is the first longitudinal study to identify that occupational group membership may play a role in the development of MetS. Longitudinal analyses showed that even after adjustment for age and health behaviors, males in seven occupational groups and females in five occupational groups had an increased risk to develop MetS. These findings indicate that occupational group matters for both men and women in the development of MetS, and suggest that differences in MetS prevalence across occupations in previous cross-sectional studies are not merely a reflection of selection of metabolically unhealthy workers into specific occupations. However, caution is warranted when interpreting the results since occupation is an aggregate exposure measure and does not show which individuals in specific jobs have, or developed, MetS. The development of MetS likely results from a combination of individual and contextual factors consisting of work-related physical activity,^29^ the psychosocial work environment,^21^ the social and physical work environment,^30^ health behaviors,^17^ and pre-existing biological factors.^31^ Disentangling the contribution of these factors in future studies may aid the development of measures to prevent MetS.

Notable differences between men and women in MetS prevalence and incidence rates were observed. Regarding baseline prevalence, only three occupational groups were associated with an increased odds for MetS in both men and women while there were three male-specific and nine female-specific occupational groups associated with an increased odds for MetS. Regarding the incidence, there was only one occupational group that was associated with an increased risk for MetS in both men and women while there were six male-specific and four female-specific occupational groups with an increased risk for MetS. In line with a previous study,^7^ we found an increased risk for MetS among males in the high-end occupational groups ‘production and specialized services managers’ and ‘information and communication technicians’, while we found no increased risk for women in similar high-end occupational groups. A Korean study also found similar results when examining different employment sectors.^32^ While the prevalence of MetS was highest among higher (29.3%) and lower (31.1%) skilled white-collar workers in men, and lowest in unskilled blue-collar workers (21.9%), the opposite was found in women. Female unskilled blue-collar (24.0%) and green-collar (24.2%) workers had the highest prevalence of MetS, while female higher skilled white-collar (16.6%) workers had the lowest prevalence.^32^ The sex differences in this and previous studies may be related to the make-up of the workforce, that is, more men or women in certain professions. Nevertheless, these findings suggest that preventive measures should differentially target men and women based on occupational group.

This study has some notable strengths. First, all components of MetS were objectively measured by trained research staff at one of the Lifelines research centers. In addition, trained research nurses recorded baseline medication use according to the ATC codes.^26^ The risk for information bias regarding MetS diagnosis was thereby limited. Second, occupational group membership was coded by Statistics Netherlands and underwent rigorous quality control by Statistics Netherlands and the study research team, and is deemed valid on the submajor group level.^24^ Third, the analyses were adjusted for age and four major health behavior factors that may confound the relationship between occupational group membership and MetS. This provides insight into the role of the occupational group separate from health behaviors that might influence MetS. Fifth, Lifelines is representative of the general population in the northern part of the Netherlands.^33^

This study also has some limitations. First, despite rigorous quality control by Statistics Netherlands on the automatic occupational coding, we cannot rule out some degree of misclassification. Our investigation of the automatic coding showed that at least 81% of participants are coded correctly on the submajor occupational level (two-digit level). In addition, we found no evidence for differential misclassification when we examined patterns of misclassification. Second, a substantial proportion of the participants at baseline did not yet complete the second follow-up measurement or were lost to follow-up, which may have induced some selection bias. Attrition analyses showed that participants without follow-up data differed significantly at baseline from participants with follow-up data regarding sociodemographic, health behavior and health-related characteristics. However, these differences were small and did not seem clinically relevant, indicating that the risk for selection bias is low. Third, MetS diagnosis during follow-up was based on physical measurements without considering medication use, as information on changes in medication was not available during follow-up. This may have resulted in missing incident MetS cases during follow-up, and thereby in an underestimation of MetS incidence. Finally, despite adjusting the analyses for four major health behavior factors, some residual confounding may be present due to the rather crude measurement of alcohol consumption and diet. Other dietary factors than fruit and vegetable intake may affect the development of MetS. In addition, unmeasured confounding might have occurred as, for example, our analyses were not adjusted for psychosocial working conditions.^21^

The study findings may have important implications for policy and practice. Non-sex-specific preventive measures and increased awareness regarding MetS are especially necessary among ‘customer services clerks’, ‘stationary plant and machine operators’, and ‘drivers and mobile plant operators’ while sex-specific approaches might be needed for other occupational groups (eg, female ‘cleaners and helpers’). Given the fact that many countries are increasing their retirement age, and MetS risk increases with age, MetS may become a serious problem in workers’ late work-life.^34^ Chronic conditions like cardiovascular disease and T2DM are likely to develop as a result from MetS,^2 17^ and are major risk factors for early work exit.^35^ Lifestyle and medication counseling programs may be beneficial for workers with MetS or with an increased risk for MetS as programs to reduce T2DM and coronary heart disease risk in individuals with an increased risk for these conditions have shown to be effective.^36 37^ Although implementation of health behavior interventions at the occupational level or worksite may pose challenges,^38^ they may be effective regarding body mass index,^39^ sedentary behavior,^40^ and eating behavior.^41^

Study findings also have important implications for researchers. To further disentangle selection and causation mechanisms, future studies need to examine the relationship between occupational group membership and MetS in a cohort of young adults entering the workforce. Such an approach may help disentangle selection and causation mechanisms since young adults entering the workforce have not been exposed to the circumstances related to their occupational group.^42^ Second, as people may change jobs and consequently occupational groups over time, their risk exposure level for developing MetS may change as well. Therefore, studies using a life course perspective, incorporating work history and job duration in analyses on the association between occupational group membership and MetS, are necessary. Third, researchers should further investigate how the workplace can be optimized as a setting for health promotion and prevention of cardiometabolic conditions like MetS.

To conclude, this study suggests that the occupational group matters for the prevalence and incidence of MetS independent of age and health behaviors, although future studies that consider other factors not included in the present analysis need to confirm our findings. The striking sex differences in the occupational distribution of MetS indicate that preventive measures should, with some exceptions, target male and female employees separately.
