# Supplementary material for: Occupational distribution of metabolic syndrome prevalence and incidence differs by sex and is not explained by age and health behavior: results from 75 000 Dutch workers from 40 occupational groups
Source: BMJ Open Diabetes Res Care. 2020 Jul 6;8(1):e001436. doi: 10.1136/bmjdrc-2020-001436 (PMC7342434; doi:10.1136/bmjdrc-2020-001436)
Supplement: Supplementary data [file bmjdrc-2020-001436supp001.pdf]

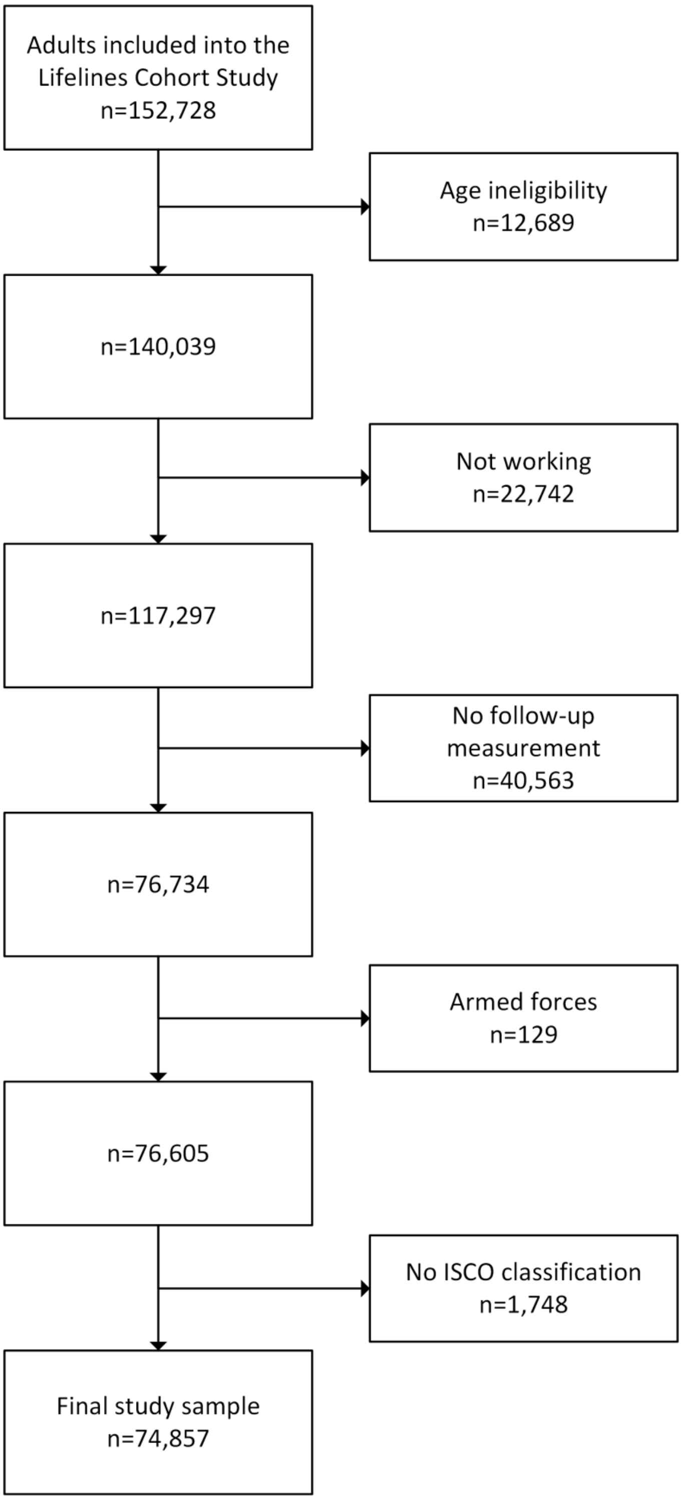

Supplemental Figure 1 Selection of the analytic study sample

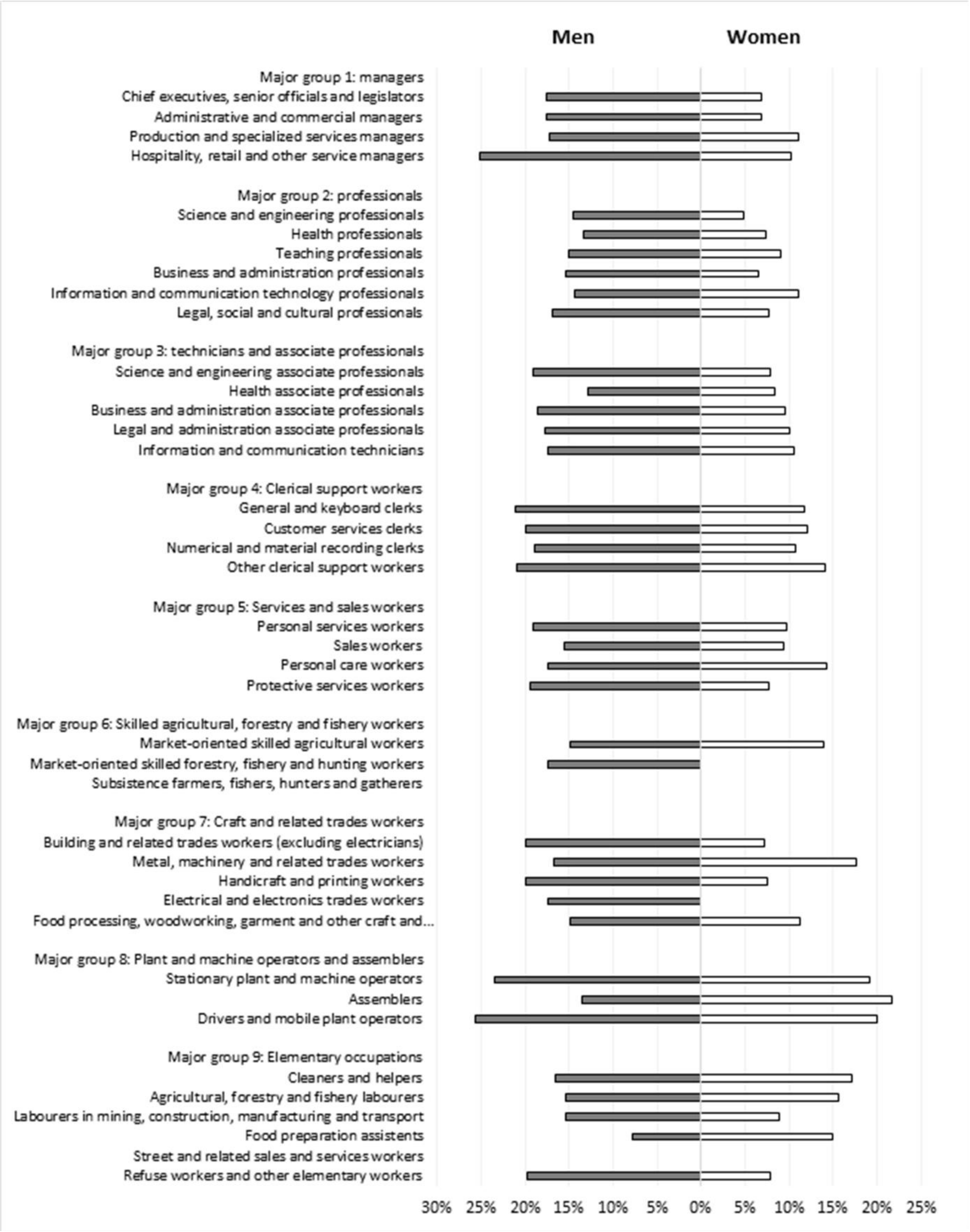

Supplemental Figure 2 Prevalence of metabolic syndrome stratified for men and women
