# Supplementary material for: Occupational distribution of metabolic syndrome prevalence and incidence differs by sex and is not explained by age and health behavior: results from 75 000 Dutch workers from 40 occupational groups
Source: BMJ Open Diabetes Res Care. 2020 Jul 6;8(1):e001436. doi: 10.1136/bmjdrc-2020-001436 (PMC7342434; doi:10.1136/bmjdrc-2020-001436)
Supplement: Supplementary data [file bmjdrc-2020-001436supp002.pdf]

Supplemental Table 1. Possible misclassification of occupations for all digit levels and specific certainty scores

Certainty score	N*	Correct classification on 1 digit	Correct classification on 2 digits	Correct classification on 3 digits	Correct classification on 4 digits
Certainty score 0-100	1403	84.0%	81.2%	79.3%	77.3%
Certainty score ≥50	949	91.3%	89.7%	88.7%	87.0%
Certainty score ≥60	791	93.6%	92.7%	92.0%	90.9%
Certainty score ≥70	600	96.7%	95.8%	95.0%	94.5%
Certainty score ≥80	448	97.5%	97.3%	96.4%	96.2%

* n=1404 of n=1432 participants were compared because n=29 participants could not be coded by Statistics Netherlands, by us, or by neither

Supplemental Table 2. Number and percentage of participants lost per major occupational group when applying certainty cut-of values of ≥50 and ≥60 for the occupational coding

Occupational group	All participants	Certainty score ≥50	% lost	Certainty score ≥60	% lost
	n	n		n	
Managers	3,675	1,536	58.2	1,155	68.6
Professionals	18,655	13,340	28.5	11,033	40.9
Technicians and associate professionals	14,688	10,198	30.6	8,534	41.9
Clerical support workers	8,834	5,605	36.5	4,468	49.4
Service and sales workers	15,182	10,472	31.0	8,186	46.1
Skilled agricultural, forestry and fishery workers	1,916	1,431	25.3	1,215	36.6
Craft and related trades workers	5,537	3,932	29.0	3,126	43.5
Plant/machine operators and assemblers	2,324	1,656	28.7	1,426	38.6
Elementary occupations	4,046	2,807	30.6	2,272	43.8

Supplemental Table 3. Comparison of baseline characteristics between participants with (n=76,734) and without (n=40,563) follow-up data

	Follow-up data		No follow-up data		P-value
	n	% or mean (SD)	n	% or mean (SD)	
**Sociodemographic factors**					
Age, y	76,734	42.5 (10.1)	40,563	39.7 (10.4)	<0.00
Sex	76,734		40,563		
Male		43.1		43.2	
Female		56.9		56.8	0.75
Educational level	75,561		39,915		
High		33.8		32.4	
Medium		42.3		42.9	
Low		23.9		24.7	<0.00
**Health behavior**					
Smoking status	72,572		36,480		
Non-smoker		47.9		47.4	
Former smoker		31.0		26.5	
Current smoker		21.1		26.2	<0.00
Alcohol consumption	76,126		37,293		
0 days p/w		19.0		18.1	
0-1 days p/w		19.7		20.8	
1-3 days p/w		40.7		41.9	
>3 days p/w		20.6		19.3	<0.00
Physical activity	73,712		35,971		
High		46.3		44.0	
Moderate		26.9		26.6	
Low		26.8		29.5	<0.00
Diet	76,115		37,286		
Healthy		21.1		17.7	
Moderate		66.6		66.3	
Unhealthy		12.2		16.0	<0.00
**Health**					
Central obesity	76,723	31.9	40,554	31.5	0.14
Raised triglycerides	76,195	17.2	37,471	17.4	0.46
Reduced HDL-cholesterol	76,195	16.8	37,470	17.8	<0.00
Raised blood pressure	76,709	38.5	40,541	35.0	<0.00
Raised fasting plasma glucose	75,877	10.6	37,171	11.0	0.03
Metabolic syndrome	76,734	13.6	40,563	12.9	0.001

Supplemental Table 4. The baseline prevalence of metabolic syndrome among men, and its association with sub-major occupational groups

				Model 1	Model 2	Model 3
		N MetS / n total	%	OR (95% CI)	OR (95% CI)	OR (95% CI)
	**Major group 2: professionals**					
21	Science and engineering professionals	206/1410	14.6	Ref	Ref	Ref
22	Health professionals	94/704	13.4	0.90 (0.69, 1.17)	0.76 (0.58, 1.00)	0.79 (0.60, 1.05)
23	Teaching professionals	194/1292	15.0	1.03 (0.83, 1.28)	**0.80 (0.64, 0.99)**	**0.78 (0.62, 0.99)**
24	Business and administration professionals	387/2508	15.4	1.07 (0.89, 1.28)	0.99 (0.82, 1.20)	0.95 (0.78, 1.16)
25	Information and communication technology professionals	228/1578	14.4	0.99 (0.80, 1.21)	1.05 (0.85, 1.29)	0.98 (0.78, 1.22)
26	Legal, social and cultural professionals	132/780	16.9	1.19 (0.94, 1.51)	1.00 (0.78, 1.28)	0.96 (0.74, 1.24)
	**Major group 1: managers**					
11	Chief executives, senior officials and legislators	58/332	17.5	1.24 (0.90, 1.70)	0.96 (0.69, 1.33)	0.82 (0.57, 1.16)
12	Administrative and commercial managers	163/927	17.6	1.25 (1.00, 1.56)	1.14 (0.91, 1.43)	1.07 (0.84, 1.36)
13	Production and specialized services managers	142/820	17.3	1.22 (0.97, 1.55)	1.03 (0.81, 1.31)	0.97 (0.75, 1.24)
14	Hospitality, retail and other service managers	31/123	25.2	**1.97 (1.28, 3.04)**	**1.83 (1.17, 2.85)**	**1.65 (1.03, 2.65)**
	**Major group 3: technicians and associate professionals**					
31	Science and engineering associate professionals	356/1867	19.1	**1.38 (1.14, 1.66)**	**1.31 (1.08, 1.59)**	**1.25 (1.02, 1.53)**
32	Health associate professionals	59/459	12.9	0.86 (0.63, 1.18)	0.78 (0.57, 1.07)	0.70 (0.50, 0.98)
33	Business and administration associate professionals	416/2231	18.6	**1.34 (1.12, 1.61)**	**1.26 (1.05, 1.52)**	1.16 (0.96, 1.42)
34	Legal and administration associate professionals	152/852	17.8	**1.27 (1.01, 1.60)**	1.20 (0.95, 1.52)	1.15 (0.90, 1.47)
35	Information and communication technicians	51/293	17.4	1.23 (0.88, 1.72)	1.22 (0.87, 1.73)	1.07 (0.75, 1.55)
	**Major group 4: Clerical support workers**					
41	General and keyboard clerks	48/227	21.1	**1.57 (1.10, 2.23)**	**1.48 (1.03, 2.12)**	1.30 (0.89, 1.91)
42	Customer services clerks	92/461	20.0	**1.46 (1.11, 1.91)**	**1.61 (1.22, 2.13)**	**1.47 (1.10, 1.98)**
43	Numerical and material recording clerks	260/1378	18.9	**1.36 (1.11, 1.66)**	**1.35(1.10, 1.65)**	1.19 (0.96, 1.47)
44	Other clerical support workers	105/502	20.9	**1.55 (1.19, 2.01)**	**1.39 (1.06, 1.82)**	1.29 (0.97, 1.71)
	**Major group 5: Services and sales workers**					
51	Personal services workers	145/758	19.1	**1.38 (1.09, 1.75)**	**1.29 (1.01, 1.64)**	1.11 (0.86, 1.43)
52	Sales workers	214/1382	15.5	1.07 (0.87, 1.32)	1.18 (0.96, 1.46)	1.05 (0.84, 1.31)
53	Personal care workers	48/276	17.4	1.23 (0.87, 1.74)	1.13 (0.79, 1.60)	1.02 (0.71, 1.48)
54	Protective services workers	148/763	19.4	**1.41 (1.11, 1.77)**	1.25 (0.99, 1.59)	1.22 (0.95, 1.56)
	**Major group 6: Skilled agricultural, forestry and fishery workers**					
61	Market-oriented skilled agricultural workers	223/1500	14.9	1.02 (0.83, 1.25)	0.91 (0.74, 1.13)	0.84 (0.68, 1.05)
62	Market-oriented skilled forestry, fishery and hunting workers	4/23	17.4	1.23 (0.41, 3.65)	1.23 (0.41, 3.75)	1.22 (0.38, 3.86)
63	Subsistence farmers, fishers, hunters and gatherers	-	-	-	-	-
	**Major group 7: Craft and related trades workers**					
71	Building and related trades workers (excluding electricians)	408/2050	19.9	**1.45 (1.21, 1.74)**	**1.43 (1.19, 1.73)**	**1.26 (1.04, 1.54)**
72	Metal, machinery and related trades workers	252/1507	16.7	1.17 (0.96, 1.43)	1.18 (0.96, 1.44)	1.07 (0.86, 1.32)

73	Handicraft and printing workers	40/200	20.0	**1.46 (1.00, 2.13)**	1.27 (0.86, 1.86)	1.16 (0.78, 1.73)
74	Electrical and electronics trades workers	98/562	17.4	1.23 (0.95, 1.61)	1.25 (0.96, 1.64)	1.07 (0.80, 1.43)
75	Food processing, woodworking, garment and other craft and related trades workers	84/562	14.9	1.03 (0.78, 1.35)	0.98 (0.74, 1.29)	0.77 (0.57, 1.05)
	**Major group 8: Plant and machine operators and assemblers**					
81	Stationary plant and machine operators	100/426	23.5	**1.79 (1.37, 2.34)**	**1.68 (1.28, 2.21)**	**1.44 (1.08, 1.93)**
82	Assemblers	12/88	13.6	0.92 (0.49, 1.73)	0.98 (0.52, 1.86)	0.79 (0.39, 1.56)
83	Drivers and mobile plant operators	368/1433	25.7	**2.02 (1.67, 2.44)**	**1.85 (1.52, 2.25)**	**1.57 (1.28, 1.93)**
	**Major group 9: Elementary occupations**					
91	Cleaners and helpers	42/253	16.6	1.16 (0.81, 1.67)	1.19 (0.82, 1.72)	1.04 (0.70, 1.54)
92	Agricultural, forestry and fishery labourers	9/59	15.3	1.05 (0.51, 2.17)	1.12 (0.53, 2.37)	1.03 (0.46, 2.28)
93	Labourers in mining, construction, manufacturing and transport	105/687	15.3	1.05 (0.82, 1.36)	1.21 (0.93, 1.57)	1.07 (0.81, 1.41)
94	Food preparation assistants	4/51	7.8	0.50 (0.18, 1.39)	0.79 (0.27, 2.31)	0.63 (0.21, 1.87)
95	Street and related sales and services workers	-	-	-	-	-
96	Refuse workers and other elementary workers	25/126	19.8	1.45 (0.91, 2.30)	1.42 (0.88, 2.29)	1.40 (0.85, 2.28)

Abbreviations: OR: odds ratio; CI: confidence interval

Model 1 is unadjusted; Model 2 is adjusted for age; Model 3 is adjusted for age, smoking, physical activity, diet, and alcohol consumption

Supplemental Table 5. The baseline prevalence of metabolic syndrome among women, and its association with sub-major occupational groups

				Model 1	Model 2	Model 3
		N MetS / n total	%	OR (95% CI)	OR (95% CI)	OR (95% CI)
	**Major group 2: professionals**					
21	Science and engineering professionals	14/287	4.9	Ref	Ref	Ref
22	Health professionals	228/3132	7.3	1.53 (0.88, 2.66)	1.28 (0.73, 2.24)	1.46 (0.78, 2.73)
23	Teaching professionals	301/3351	9.0	**1.92 (1.11, 3.33)**	1.46 (0.84, 2.54)	1.63 (0.88, 3.04)
24	Business and administration professionals	128/1936	6.6	1.38 (0.78, 2.43)	1.20 (0.68, 2.12)	1.36 (0.72, 2.57)
25	Information and communication technology professionals	29/264	11.0	**2.41 (1.24, 4.66)**	**2.04 (1.05, 3.97)**	2.08 (0.99, 4.37)
26	Legal, social and cultural professionals	107/1389	7.7	1.63 (0.92, 2.88)	1.38 (0.77, 2.45)	1.61 (0.85, 3.06)
	**Major group 1: managers**					
11	Chief executives, senior officials and legislators	9/133	6.8	1.41 (0.60, 3.36)	1.09 (0.46, 2.60)	1.15 (0.45, 2.98)
12	Administrative and commercial managers	28/412	6.8	1.42 (0.73, 2.75)	1.12 (0.58, 2.48)	1.23 (0.59, 2.57)
13	Production and specialized services managers	38/346	11.0	**2.41 (1.28, 4.54)**	1.75 (0.92, 3.33)	1.96 (0.96, 3.99)
14	Hospitality, retail and other service managers	10/98	10.2	2.22 (0.95, 5.16)	1.74 (0.74, 4.11)	1.85 (0.74, 4.62)
	**Major group 3: technicians and associate professionals**					
31	Science and engineering associate professionals	21/266	7.9	1.67 (0.83, 3.36)	1.47 (0.73, 3.00)	1.38 (0.63, 3.00)
32	Health associate professionals	234/2803	8.3	**1.78 (1.02, 3.09)**	1.50 (0.86, 2.62)	1.61 (0.86, 3.01)
33	Business and administration associate professionals	285/2967	9.6	**2.07 (1.19, 3.59)**	1.74 (1.00, 3.02)	1.83 (0.98, 3.41)
34	Legal and administration associate professionals	285/2846	10.0	**2.17 (1.25, 3.76)**	**1.86 (1.06, 3.23)**	**1.96 (1.05, 3.66)**
35	Information and communication technicians	11/104	10.6	**2.31 (1.01, 5.26)**	1.98 (0.86, 4.56)	1.97 (0.80, 4.86)
	**Major group 4: Clerical support workers**					
41	General and keyboard clerks	240/2057	11.7	**2.58 (1.48, 4.48)**	**1.95 (1.12, 3.41)**	1.86 (0.99, 3.48)
42	Customer services clerks	182/1512	12.0	**2.67 (1.53, 4.67)**	**2.14 (1.22, 3.76)**	**2.22 (1.18, 4.18)**
43	Numerical and material recording clerks	175/1622	10.8	**2.36 (1.35, 4.13)**	**1.85 (1.05, 3.25)**	1.87 (0.99, 3.51)
44	Other clerical support workers	149/1059	14.1	**3.19 (1.82, 5.61)**	**2.50 (1.42, 4.42)**	**2.33 (1.23, 4.41)**
	**Major group 5: Services and sales workers**					
51	Personal services workers	224/2291	9.8	**2.11 (1.21, 3.68)**	**1.79 (1.02, 3.14)**	1.74 (0.93, 3.27)
52	Sales workers	340/3609	9.4	**2.03 (1.17, 3.51)**	**1.84 (1.06, 3.20)**	1.77 (0.95, 3.31)
53	Personal care workers	822/5792	14.2	**3.22 (1.87, 5.55)**	**2.34 (1.36, 4.05)**	**2.22 (1.20, 4.11)**
54	Protective services workers	24/310	7.7	1.64 (0.83, 3.23)	1.53 (0.77, 3.03)	1.53 (0.72, 3.25)
	**Major group 6: Skilled agricultural, forestry and fishery workers**					
61	Market-oriented skilled agricultural workers	55/392	14.0	**3.18 (1.73, 5.85)**	**2.21 (1.15, 3.94)**	**2.21 (1.11, 4.37)**
62	Market-oriented skilled forestry, fishery and hunting workers	-	-	-	-	-
63	Subsistence farmers, fishers, hunters and gatherers	-	-	-	-	-
	**Major group 7: Craft and related trades workers**					
71	Building and related trades workers (excluding electricians)	5/69	7.2	1.52 (0.53, 4.38)	1.10 (0.38, 3.21)	1.40 (0.46, 4.27)
72	Metal, machinery and related trades workers	9/51	17.6	**4.18 (1.70, 10.3)**	**3.00 (1.20, 7.46)**	**3.56 (1.35, 9.39)**

73	Handicraft and printing workers	6/79	7.6	1.60 (0.59, 4.32)	1.29 (0.47, 3.51)	1.09 (0.33, 3.58)
74	Electrical and electronics trades workers	-	-	1.77 (0.21, 14.7)	-	-
75	Food processing, woodworking, garment and other craft and related trades workers	38/335	11.3	**2.49 (1.32, 4.70)**	**2.08 (1.10, 3.96)**	2.00 (0.99, 4.09)
	**Major group 8: Plant and machine operators and assemblers**					
81	Stationary plant and machine operators	22/115	19.1	**4.61 (2.27, 9.38)**	**3.75 (1.83, 7.69)**	**3.44 (1.57, 7.54)**
82	Assemblers	10/46	21.7	**5.42 (2.24, 13.1)**	**4.33 (1.77, 10.6)**	**3.43 (1.26, 9.33)**
83	Drivers and mobile plant operators	43/215	20.0	**4.87 (2.59, 9.18)**	**3.28 (1.73, 6.23)**	**2.92 (1.43, 5.95)**
	**Major group 9: Elementary occupations**					
91	Cleaners and helpers	385/2244	17.2	**4.04 (2.33, 6.99)**	**2.71 (1.56, 4.71)**	**2.30 (1.24, 4.29)**
92	Agricultural, forestry and fishery labourers	11/70	15.7	**3.64 (1.57, 8.41)**	**3.13 (1.33, 7.37)**	**2.69 (1.03, 7.01)**
93	Labourers in mining, construction, manufacturing and transport	26/291	8.9	1.91 (0.98, 3.74)	1.86 (0.94, 3.66)	1.35 (0.62, 2.93)
94	Food preparation assistants	25/168	14.9	**3.41 (1.72, 6.76)**	**3.25 (1.62, 6.53)**	**2.57 (1.16, 5.69)**
95	Street and related sales and services workers	-	-	-	-	-
96	Refuse workers and other elementary workers	7/89	7.9	1.66 (0.65, 4.26)	1.33 (0.51, 3.47)	0.86 (0.28, 2.60)

Abbreviations: OR: odds ratio; CI: confidence interval

Model 1 is unadjusted; Model 2 is adjusted for age; Model 3 is adjusted for age, smoking, physical activity, diet, and alcohol consumption
